# Foodborne Illness Outbreaks at Retail Establishments — National Environmental Assessment Reporting System, 16 State and Local Health Departments, 2014–2016

**DOI:** 10.15585/mmwr.ss6801a1

**Published:** 2019-02-22

**Authors:** Lauren E. Lipcsei, Laura G. Brown, Erik W. Coleman, Adam Kramer, Matthew Masters, Beth C. Wittry, Kirsten Reed, Vincent J. Radke

**Affiliations:** 1 Division of Environmental Health Science and Practice, National Center for Environmental Health, CDC

## Abstract

**Problem/Condition:**

State and local public health departments report hundreds of foodborne illness outbreaks each year to CDC and are primarily responsible for investigations of these outbreaks. Typically, investigations involve epidemiology, laboratory, and environmental health components. Health departments voluntarily report epidemiologic and laboratory data from their foodborne illness outbreak investigations to CDC through the Foodborne Disease Outbreak Surveillance System (FDOSS); however, minimal environmental health data from outbreak investigations are reported to FDOSS.

**Period Covered:**

2014–2016.

**Description of System:**

In 2014, CDC launched the National Environmental Assessment Reporting System (NEARS) to complement FDOSS surveillance and to use these data to enhance prevention efforts. State and local health departments voluntarily report data from their foodborne illness outbreak investigations of retail food establishments. These data include characteristics of foodborne illness outbreaks (e.g., agent), characteristics of establishments with outbreaks (e.g., number of meals served daily), food safety policies and practices of these establishments (e.g., glove use policies), and characteristics of outbreak investigations (e.g., timeliness of investigation activities). NEARS is the only available data source that includes characteristics of retail establishments with foodborne illness outbreaks.

**Results:**

During 2014–2016, a total of 16 state and local public health departments reported data to NEARS on 404 foodborne illness outbreaks at retail establishments. The majority of outbreaks with a suspected or confirmed agent were caused by norovirus (61.1%). The majority of outbreaks with identified contributing factors had at least one factor associated with food contamination by a worker who was ill or infectious (58.6%). Almost half (47.4%) of establishments with outbreaks had a written policy excluding ill workers from handling food or working. Approximately one third (27.7%) had a written disposable glove use policy. Paid sick leave was available for at least one worker in 38.3% of establishments. For most establishments with outbreaks (68.7%), environmental health investigators initiated their component of the investigation soon after learning about the outbreak (i.e., the same day) and completed their component in one or two visits to the establishment (75.0%). However, in certain instances, contacting the establishment and completing the environmental health component of the investigation occurred much later (>8 days).

**Interpretation:**

Most outbreaks reported to NEARS were caused by norovirus, and contamination of food by workers who were ill or infectious contributed to more than half of outbreaks with contributing factors; these findings are consistent with findings from other national outbreak data sets and highlight the role of workers in foodborne illness outbreaks. The relative lack of written policies for ill workers and glove use and paid sick leave for workers in establishments with outbreaks indicates gaps in food safety practices that might have a role in outbreak prevention. The environmental health component of the investigation for most outbreaks was initiated quickly, yet the longer initiation timeframe for certain outbreaks suggests the need for improvement.

**Public Health Action:**

Retail establishments can reduce viral foodborne illness outbreaks by protecting food from contamination through proper hand hygiene and excluding workers who are ill or infectious from working. NEARS data can help prioritize training and interventions for state and local food safety programs and the retail food establishment industry by identifying gaps in food safety policies and practices and types of establishments vulnerable to outbreaks. Improvement of certain outbreak investigation practices (e.g., delayed initiation of environmental health investigations) can accelerate identification of the agent and implementation of interventions. Future analysis comparing establishments with and without outbreaks will contribute knowledge about how establishments’ characteristics and food safety policies and practices relate to foodborne illness outbreaks and provide information to develop effective prevention approaches.

## Introduction

Public health departments report hundreds of outbreaks each year to CDC. During 2009–2015, state, local, and territorial health departments reported 5,760 foodborne illness outbreaks to CDC (*1*). Most of these outbreaks occurred in retail food establishments (*1*).

State and local public health departments are typically responsible for regulating and ensuring food safety in retail food establishments. They do this primarily through inspecting establishments to ensure they comply with their jurisdictions’ food safety regulations. The U.S. Food and Drug Administration (FDA) Food Code is the basis of most jurisdictions’ food safety regulations. The FDA Food Code is a model set of science-based, comprehensive food safety regulations intended to reduce foodborne illness risk in retail food establishments (*2*). For example, the Food Code includes guidelines that

- limit opportunities for food workers to contaminate food, such as prohibiting workers who are ill or infectious from working with food and prohibiting workers from handling ready-to-eat food (i.e., foods that need no further preparation) with their bare hands (e.g., through glove use); and
- require kitchen managers to be certified in food safety (i.e., pass a food safety knowledge test administered by an accredited program).

State and local public health departments also investigate foodborne illness outbreaks. Data from these investigations provide insights into the epidemiology of foodborne illness, such as identifying the pathogens and foods that lead to illness (*1*). This information can be used to help prevent foodborne illness outbreaks and sporadic foodborne illnesses that can have the same epidemiologic profile as outbreaks.

State and local public health departments provide epidemiologic and laboratory data from their investigations to CDC through the Foodborne Disease Outbreak Surveillance System (FDOSS) (*3*). Typically, epidemiology or communicable disease control programs within health departments collect and report these data, which include the etiologic agent; food; setting; and number of illnesses, hospitalizations, and deaths associated with an outbreak. These data have led to discoveries of new and emerging foodborne illness agents and specific agent-food pairs (*4*).

Environmental health programs within state and local health departments also are involved in investigations of foodborne illness outbreaks. The environmental health component of the investigation, or environmental assessment, describes how the environment contributed to the introduction or transmission of agents that caused illness. During these assessments, environmental health investigators typically interview the manager of the establishment with an outbreak about characteristics such as food preparation policies and practices that might have contributed to the outbreak. Environmental health investigators also review the processes used in preparing food items suspected in the outbreak and observe workers’ food preparation practices. After all investigation activities are completed, the epidemiologic, laboratory, and environmental health information is reviewed to determine the outbreak contributing factors, which are the conditions that enabled or amplified a foodborne illness outbreak. These factors can contribute to contamination of food with foodborne illness agents, proliferation of microbial agents in food, or survival of foodborne illness agents in food after a process that should have eliminated or reduced them.

Although FDOSS captures data on foodborne illness outbreak contributing factors, the system does not capture most other environmental assessment data and is not limited to retail food establishments. These data about the context in which outbreaks occur are important to outbreak prevention. For example, data on worker practices associated with outbreaks can provide information about interventions that encourage retail food establishments to improve worker practices. Because of the importance of these environmental assessment data, CDC developed the National Environmental Assessment Reporting System (NEARS) to capture data from health departments’ environmental assessments of outbreaks (*5*). NEARS was designed to be a complementary surveillance system to FDOSS. The Environmental Health Specialists Network (EHS-Net), a CDC-funded network of environmental health specialists and epidemiologists from CDC, FDA, the U.S. Department of Agriculture, and multiple state and local health departments (*6*), helped develop NEARS.

This report summarizes selected data reported to NEARS for foodborne illness outbreaks that occurred during 2014–2016. The data describe the outbreaks, the establishments where the outbreaks occurred, including their food safety policies, and the outbreak investigations. State and local public health departments responsible for ensuring food safety and investigating foodborne illness outbreaks can use these data to help identify gaps in their outbreak investigation practices and in retail food establishment policies.

## Methods

### Description of the System and Case Definition

The majority of foodborne illness outbreaks occur in retail food establishments (i.e., places that prepare and serve food to consumers) (*1*). In 2014, NEARS was launched to collect data on outbreaks associated with such establishments (*4*). CDC defines a foodborne illness outbreak as an incident in which two or more persons experience a similar illness resulting from the ingestion of a common food (*7*); most state and local health departments have a similar definition. Outbreak agents were classified as confirmed if they were laboratory confirmed according to CDC laboratory and clinical guidelines (*7*); otherwise they were classified as suspected. During 2014–2016, a total of 16 state and local health departments (California; Coconino County, Arizona; Connecticut; Davis County, Utah; Fairfax County, Virginia; Harris County, Texas; Michigan; Minnesota; New York City; New York State; Rhode Island; South Carolina; Southern Nevada Health District; Tennessee; Washington; and Wisconsin) reported environmental assessment data to NEARS from at least one foodborne illness outbreak occurring in a retail food establishment. Supplementary data on foodborne illness outbreaks reported to NEARS (https://stacks.cdc.gov/view/cdc/61382) and retail establishments with outbreaks (https://stacks.cdc.gov/view/cdc/61383) are available.

NEARS complements FDOSS by collecting data from state and local foodborne illness outbreak investigations that are not collected in FDOSS. Although some data points are collected in both systems (e.g., outbreak agent), this redundancy is designed to ensure that outbreaks can be matched accurately across the two systems.

### Data Sources, Collection, and Variables

NEARS data sources include environmental health investigators and their epidemiology and laboratory counterparts, as well as interviews with establishment managers (Box 1). After each foodborne illness outbreak investigation in a retail food establishment is completed, participating health departments voluntarily report their environmental health investigation data to CDC through the NEARS online data management system on CDC’s website. Environmental health investigators’ epidemiologic and laboratory counterparts provide the data on outbreak characteristics. The environmental assessments provide data on characteristics and policies of establishments with outbreaks, primarily through interviews with managers. The environmental health investigators determine outbreak investigation characteristics. Not all data points are collected during all investigations; thus, denominators vary throughout the results.

BOX 1 **Data sources for characteristics of foodborne illness outbreaks, characteristics and policies of retail establishments with outbreaks, and characteristics of investigations — National Environmental Assessment Reporting System, 2014–2016**

**Table:** 

**Data collected**	**Source**
**Outbreak characteristics**	
Primary agent identification — confirmed (laboratory-confirmed by laboratory and clinical guidelines) or suspected (not confirmed by the guidelines) (In 2014, these data were obtained from the Foodborne Disease Outbreak Surveillance System; during 2015–2016, environmental health investigators reported these data to the National Environmental Assessment Reporting System)	Epidemiology and laboratory investigation counterparts
Contributing factor identification (factors that contribute to the contamination, proliferation, and survival of foodborne illness agents on food)	Investigation team determination
Outbreak also reported to the Foodborne Disease Outbreak Surveillance System	Epidemiology and laboratory investigation counterparts
**Outbreak establishment characteristics**	
Ownership — independent or chain (establishment shares name and operations with at least one other establishment)	Establishment manager interview
Establishment type — restaurant (fixed establishment that prepares and serves food to customers) or other (e.g., institutions, mobile food units, temporary food stands, or restaurants in supermarkets, etc.)	Environmental health investigator determination
Average number of meals served daily	Establishment manager interview
Most complex food preparation process • Complex — food item requires a pathogen kill step (a process, such as cooking or freezing, that reduces or eliminates pathogens) and holding beyond same-day service, or a kill step and some combination of holding, cooling, reheating, and freezing • Cook-serve — food item is prepared for same-day service; at least one involves a kill step such as cooking • Prep-serve — food item is prepared and served without a kill step	Environmental health investigator determination
Menu type (e.g., American or Indian)	Environmental health investigator determination
Number of critical violations on previous inspection (i.e., violations of regulations that help eliminate or reduce hazards associated with foodborne illness; also called priority or priority foundation items)	Environmental health investigator determination
**Outbreak establishment policies**	
Policy requiring workers to tell their manager when they are ill	Establishment manager interview
Policy restricting or excluding ill workers from working	Establishment manager interview
Paid sick leave available for at least one worker	Establishment manager interview
Disposable glove use policy	Establishment manager interview
Disposable glove use policy requiring food workers to wear gloves at all times when working in the kitchen, when handling ready-to-eat food, and when they have cuts or other skin injuries	Establishment manager interview
Kitchen manager food safety certification requirement	Establishment manager interview
**Outbreak investigation characteristics**	
Number of visits to the establishment with an outbreak to complete environmental assessment	Environmental health investigator determination
Number of days between identification of establishment for an environmental assessment and first contact with the establishment, observation, and manager interview	Environmental health investigator determination

Data are collected and presented on four sets of variables: characteristics of foodborne illness outbreaks, characteristics of establishments with outbreaks, policies of establishments with outbreaks, and characteristics of investigations.

- **Outbreak characteristics***.* Characteristics include the outbreak agent and contributing factors. FDA and CDC have identified 32 outbreak contributing factors, divided into three groups (*8*):
  - contamination of food with a foodborne illness agent;
  - proliferation or growth of microbial agents in food (proliferation can mean an increase in the number of bacteria, the production of toxins, or both); and
  - survival of foodborne illness agents after a process, such as cooking, that should have eliminated or reduced them.
- **Outbreak establishment characteristics.** Characteristics that have been hypothesized or found to be associated with retail food establishment food safety. These include ownership (independent or chain [shares name and operation with at least one other establishment]) and number of meals served daily (*9*–*12*).
- **Outbreak establishment policies.** Policies recommended by FDA in the Food Code to reduce foodborne illness risk. These include limiting opportunities for food workers to contaminate food, such as prohibiting workers who are ill or infectious from working with food and prohibiting workers from handling ready-to-eat food (i.e., foods that need no further preparation) with their bare hands (e.g., through glove use), and requiring kitchen managers to be certified in food safety (i.e., pass a food safety knowledge test administered by an accredited program). Data also are included on the availability of paid sick leave for ill workers. Although the Food Code specifically does not recommend paid sick leave, the food service industry could explore this policy as a potential method to help keep ill workers from working (*13*).
- **Outbreak investigation characteristics.** Characteristics that have been hypothesized or found to be associated with investigation effectiveness, such as the timeliness of outbreak environmental assessments (*14*,*15*).

### Data Analysis

CDC calculated descriptive statistics on four sets of NEARS variables. These were characteristics of foodborne illness outbreaks, characteristics of establishments with outbreaks, policies of establishments with outbreaks, and characteristics of investigations.

## Results

During 2014–2016, state and local health departments reported 404 foodborne illness outbreaks in retail establishments to NEARS. Of these, 111 (27.5%) occurred in 2014, 113 (28.0%) in 2015, and 180 (44.6%) in 2016. A total of 384 (95.0%) of these outbreaks occurred in one location, and 20 (5.0%) occurred in multiple locations. Data were reported to NEARS on 415 establishments with outbreaks. Most (83.7%, 338 of 404) outbreaks reported to NEARS also were reported to FDOSS. This percentage is expected to increase in the future because of updates to the reporting system and improvements in linking processes. A supplementary summary report is available (https://www.cdc.gov/nceh/ehs/nears/outbreak-investigations-restaurants-2014-16.html).

### Outbreak Characteristics

Investigations identified an agent in 311 (77.0%) outbreaks (Table 1). Of these agents, 31.8% were suspected and 68.2% were confirmed. Most identified agents were viral (61.7%), followed by bacterial (34.4%) and toxic, chemical, or other (3.9%). Overall, norovirus was the most common agent, accounting for 61.1% of outbreaks where an agent was identified. The second most common agent was *Salmonella*, accounting for 16.1% of outbreaks with an identified agent.

**TABLE 1:** Foodborne illness outbreaks with a suspected or confirmed identified agent — National Environmental Assessment Reporting System, 16 state and local health departments, 2014–2016

Agent	Suspected	Confirmed	Total
	No. (%)*	No. (%)*	No. (%)*
**Virus**			
Norovirus	66 (21.2)	124 (39.9)	**190 (61.1)**
Hepatitis A	0 (0.0)	2 (0.6)	**2 (0.6)**
**Total viral outbreaks**	**66 (21.2)**	**126 (40.5)**	**192 (61.7)**
**Bacteria**			
*Salmonella* species	2 (0.6)	48 (15.4)	**50 (16.1)**
*Clostridium perfringens*	9 (2.9)	8 (2.6)	**17 (5.5)**
*Campylobacter* species	2 (0.6)	9 (2.9)	**11 (3.5)**
*Bacillus cereus*	5 (1.6)	1 (0.3)	**6 (1.9)**
*Escherichia coli O157:H7/STEC*	0 (0.0)	10 (3.2)	**10 (3.2)**
*Staphylococcus aureus*	5 (1.6)	2 (0.6)	**7 (2.3)**
*Shigella* species	0 (0.0)	2 (0.6)	**2 (0.6)**
*Vibrio* species	1 (0.3)	2 (0.6)	**3 (1.0)**
*Listeria monocytogenes*	0 (0.0)	1 (0.3)	**1 (0.3)**
*Yersinia* species	0 (0.0)	0 (0.0)	**0 (0.0)**
**Total bacterial outbreaks**	**24 (7.7)**	**83 (26.7)**	**107 (34.4)**
**Toxin, chemical, and other^†^**			
Scombroid toxin	4 (1.3)	1 (0.3)	**5 (1.6)**
Ciguatoxin	1 (0.3)	0 (0.0)	**1 (0.3)**
Chemical	1 (0.3)	0 (0.0)	**1 (0.3)**
Other	3 (1.0)	2 (0.6)	**5 (1.6)**
**Total toxin outbreaks**	**9 (2.9)**	**3 (1.0)**	**12 (3.9)**
**Total outbreaks**	**99 (31.8)**	**212 (68.2)**	**311 (100.0)**

**Abbreviation:** STEC = Shiga toxin–producing *Escherichia coli*.

* All numbers are divided by the total number of outbreaks with a suspected or confirmed agent (denominator = 311) to obtain the percentage. Because of rounding, some percentages might not total 100%.

^†^ Toxins produced by bacteria are included in the bacteria category; natural toxins, such as marine and mushroom, are included in the toxin category.

Investigators identified at least one contributing factor in 251 (62.1%) outbreaks. Outbreaks can have more than one contributing factor, and 455 were identified. Of the 251 outbreaks with an identified contributing factor, 214 (85.3%) had at least one contamination factor, 69 (27.5%) had at least one proliferation factor, and 44 (17.5%) had at least one survival factor (Table 2). The top three contributing factors were related to food contamination by an ill worker; 147 (58.6%) outbreaks with an identified contributing factor had at least one of these factors.

**TABLE 2:** Factors contributing to foodborne illness outbreaks, by type of factor — National Environmental Assessment Reporting System, 16 state and local health departments, 2014–2016

Contributing factor	No. (%)*
**Contamination of food with a foodborne illness agent**	
Bare-hand contact by a food handler, worker, or preparer who was suspected to have an infectious illness (C10 )	70 (27.9)
Other mode of contamination (excluding cross-contamination) by a food handler, worker, or preparer who was suspected to have an infectious illness (C12)	58 (23.1)
Glove-hand contact by a food handler, worker, or preparer who was suspected to have an infectious illness (C11)	39 (15.5)
Cross-contamination of ingredients (does not include ill food workers) (C9)	28 (11.2)
Contaminated raw product — food was intended to be consumed raw or undercooked or underprocessed (C7)	15 (6.0)
Other source of contamination (C15)	24 (9.6)
Contaminated raw product — food was intended to be consumed after a kill step (C6)	14 (5.6)
Toxic substance part of the tissue (e.g., ciguatera) (C1)	5 (2.0)
Foods contaminated by nonfood handler, worker, or preparer who was suspected to have an infectious illness (C13)	9 (3.6)
Poisonous substance accidentally or inadvertently added (C3)	1 (0.4)
Foods originating from sources shown to be contaminated or polluted (C8)	4 (1.6)
Poisonous substance intentionally or deliberately added (C2)	0 (0.0)
Addition of excessive quantities of ingredients that are toxic in large amounts (e.g., niacin poisoning in bread) (C4)	0 (0.0)
Toxic container (e.g., galvanized containers with acid foods) (C5)	0 (0.0)
Storage in contaminated environment (C14)	13 (5.2)
**Total contamination factors**	**280 (100.0)**
**Proliferation or growth of microbial agents in food (increase in number of bacteria or the production of toxins)**	
Improper or slow cooling (P8)	25 (10.0)
No attempt to control the temperature of implicated food or the length of time food was out of temperature control (during food service or display of food) (P2)	23 (9.2)
Improper cold holding due to malfunctioning refrigeration equipment (P4)	13 (5.2)
Improper hot holding due to an improper procedure or protocol (P7)	14 (5.6)
Improper cold holding due to an improper procedure or protocol (P5)	18 (7.2)
Food preparation practices that support proliferation of pathogens (during food preparation) (P1)	18 (7.2)
Improper hot holding due to malfunctioning equipment (P6)	5 (2.0)
Inadequate modified atmosphere packaging (e.g., vacuum-packed fish) (P10)	2 (0.8)
Improper adherence to approved plan for using time as a public health control (P3)	1 (0.4)
Prolonged cold storage (P9)	0 (0.0)
Inadequate processing (e.g., acidification, water activity, or fermentation) (P11)	1 (0.4)
Other situations that promoted or allowed microbial growth or toxin production (P12)	2 (0.8)
**Total proliferation factors**	**122 (100.0)**
**Survival of foodborne illness agents after a process, such as cooking, that should have eliminated or reduced them**	
Insufficient time, temperature, or both during cooking or heat processing (e.g., roasted poultry, canned foods, or pasteurization) (S1)	27 (10.8)
Insufficient time, temperature, or both during reheating (S2)	12 (4.8)
Insufficient time, temperature control, or both during freezing (S3)	0 (0.0)
Insufficient or improper use of chemical processes designed for pathogen destruction (S4)	10 (4.0)
Other process failures that permit agent survival (S5)	4 (1.6)
**Total survival factors**	**53 (100.0)**
**Total contributing factors**	**455 (100.0)**

**Source:** CDC [Internet]. NORS guidance for contributing factors (CF) in foodborne outbreak reports. Atlanta, GA: US Department of Health and Human Services, CDC; 2018. https://www.cdc.gov/nors/downloads/appendix-d.pdf

**Abbreviations**: C = contamination; P = proliferation; S = survival.

* Denominator = 251; some outbreaks had more than one identified contributing factor, so percentages sum to more than 100%. These designations (e.g., C1, P6, or S2) are used by outbreak investigators to refer to the type of contributing factor (e.g., contamination, proliferation, or survival) and its numerical position on the contributing factor list.

All three types of contributing factors (i.e., contamination of food with agents, proliferation of agents, and survival of agents) were represented among the top 10 contributing factors to foodborne illness outbreaks (Box 2). The most common contributing factor (27.9%) was bare-hand contact by a food worker suspected to have an infectious illness, followed by contamination through a method other than hand contact by a food worker suspected to have an infectious illness (23.1%) and glove-hand contact by a food worker suspected to have an infectious illness (15.5%) (Table 2). The most common proliferation and survival contributing factors were improper or slow cooling of hot food (10.0%) and insufficient time or temperature during cooking or heat processing (10.8%).

BOX 2 Top 10 contributing factors to foodborne illness outbreaks,* by type — National Environmental Assessment Reporting System, 2014–2016

**Contamination of food with a foodborne illness agent**

Bare-hand contact by a food handler, worker, or preparer with a suspected infectious illness

Other mode of contamination (excluding cross-contamination) by a food handler, worker, or preparer with a suspected infectious illness

Glove-hand contact by a food handler, worker, or preparer with a suspected infectious illness

Cross-contamination of ingredients

Other source of contamination

Contaminated raw product — food was intended to be consumed raw or undercooked or underprocessed

Contaminated raw product — food was intended to be consumed after a kill step

**Proliferation or growth of microbial agents in food (increase in number of bacteria or the production of toxins)**

Improper or slow cooling

No attempt was made to control the temperature of implicated food or the length of time food was out of temperature control

**Survival of foodborne illness agents after a process, such as cooking, that should have eliminated or reduced them**

Insufficient time, temperature, or both during cooking or heat processing

### Outbreak Establishment Characteristics

Most establishments with outbreaks were independently owned (72.9%, 237 of 325), were restaurants (80.2%, 333 of 415), and served complex food items (i.e., a food item required a kill step, which is a process, such as cooking, that reduces or eliminates foodborne illness pathogens, and other food preparation processes, such as cooling and reheating) (87.2%, 362 of 415) (Table 3). More than half of establishments with outbreaks (54.6%, 161 of 295) served ≤200 meals daily (upper range: 7,500). The most common menu type was American (nonethnic) (55.9%, 232 of 415), and most (65.8%) establishments with outbreaks received one or more critical violations on their last routine inspection before the outbreak.

**TABLE 3:** Characteristics of retail establishments with foodborne illness outbreaks — National Environmental Assessment Reporting System, 16 state and local health departments, 2014–2016

Establishment characteristic	No. (%)*
**Ownership**	
Independent	237 (72.9)
Chain	88 (27.1)
**Total**	**325 (100.0)**
**Establishment type**	
Restaurant	333 (80.2)
Other	82 (19.8)
**Total**	**415 (100.0)**
**Most complex food preparation process**	
Complex — food item requires a pathogen kill step (a process, such as cooking or freezing, that reduces or eliminates pathogens) and holding beyond same-day service, or a kill step and some combination of holding, cooling, reheating, and freezing	362 (87.2)
Cook-serve — food item is prepared for same-day service; at least one involves a kill step such as cooking	39 (9.4)
Prep-serve — food item is prepared and served without a kill step	14 (3.4)
**Total**	**415 (100.0)**
**Number of meals served daily**	
<100	88 (29.8)
101–200	73 (24.7)
201–300	48 (16.3)
301–400	29 (9.8)
401–500	14 (4.8)
501–7,500	43 (14.6)
**Total**	**295 (100.0)**
**Menu**	
American	232 (55.9)
Other (e.g., Mediterranean, Indian, or Spanish)	72 (17.3)
Mexican	38 (9.2)
Italian	30 (7.2)
Chinese	23 (5.5)
Japanese	16 (3.9)
Thai	4 (1.0)
**Total**	**415 (100.0)**
**Critical violations on last inspection**	
None	142 (34.2)
>1	273 (65.8)
**Total**	**415 (100.0)**

* Denominators vary because of missing data. Because of rounding, some percentages might not total 100%.

### Outbreak Establishment Policies

More than half of establishments with outbreaks (56.3%, 179 of 318) had a written policy and 36.2% (115) had a verbal policy requiring food workers to notify their manager when they were ill (Table 4). About half (47.4%, 144 of 304) of establishments had a written policy and 39.1% had a verbal policy that prevented ill workers from handling food (i.e., restriction) or prevented ill workers from working (i.e., exclusion). In 118 of 308 (38.3%) establishments, paid sick leave was available for at least one food worker. The majority of establishments with outbreaks (62.3%, 198 of 318) had a verbal policy concerning disposable glove use; an additional 27.7% had a written disposable glove use policy. Glove use policy requirements were varied. Most establishments required food workers to wear gloves when handling ready-to-eat foods (97.2%, 278 of 286) and when they had cuts or other skin injuries (98.6%, 278 of 282), and half (49.7%, 142 of 286) required food workers to wear gloves at all times when working in the kitchen. In 243 of 314 (77.4%) establishments, kitchen managers were required to have a food safety certification.

**TABLE 4:** Policies of retail establishments with foodborne illness outbreaks — National Environmental Assessment Reporting System, 16 state and local health departments, 2014–2016

Establishment policy	No. (%)*
**Policy requiring food workers to tell their manager when they are ill**	
Yes	115 (36.2)
Yes, and it’s written	179 (56.3)
No	24 (7.5)
**Total**	**318 (100.0)**
**Policy restricting or excluding ill workers from working**	
Yes	119 (39.1)
Yes, and it’s written	144 (47.4)
No	41 (13.5)
**Total**	**304 (100.0)**
**Paid sick leave available for at least one worker**	
Yes	118 (38.3)
No	190 (61.7)
**Total**	**308 (100.0)**
**Disposable glove use policy**	
Yes	198 (62.3)
Yes, and it’s written	88 (27.7)
No	32 (10.1)
**Total**	**318 (100.0)**
**Glove use policy requiring food workers to wear gloves when handling ready-to-eat food^†^**	
Yes	278 (97.2)
No	8 (2.8)
**Total**	**286 (100.0)**
**Glove use policy requiring food workers to wear gloves when they have cuts or other skin injuries^†^**	
Yes	278 (98.6)
No	4 (1.4)
**Total**	**282 (100.0)**
**Glove use policy requiring food workers to wear gloves at all times when working in the kitchen^†^**	
Yes	142 (49.7)
No	144 (50.3)
**Total**	**286 (100.0)**
**Kitchen manager food safety certification requirement**	
Yes	243 (77.4)
No	71 (22.6)
**Total**	**314 (100.0)**

* Denominators vary because of missing data and interview skip patterns. Because of rounding, some percentages might not total 100%.

^†^ Only asked if the manager said they have a glove use policy.

### Outbreak Investigation Characteristics

Three fourths (74.6%, 306 of 410) of environmental assessments were completed in one or two visits to the establishment; the remaining assessments were completed in three or more visits (Table 5). Investigators contacted most (68.7%, 285 of 415) of the establishments with outbreaks the same day they were identified for an environmental assessment. The mode of contact varied (e.g., telephone, e-mail, or in person). For the remaining establishments, contact occurred 1–2 days (23.4%, 97 of 415) and ≥3 days (7.9%, 9 of 415) after identification. Half (49.6%, 175 of 353) of observations were conducted the same day the establishment was identified for an environmental assessment. The remaining observations were conducted 1–2 days after identification (28.0%) and ≥3 days after identification (22.4%). One fourth (25.8%, 82 of 318) of interviews with managers were conducted the same day the establishment was identified for an assessment. The remaining interviews were conducted 1–2 days (19.5%), 3–7 days (12.9%), and ≥8 days (41.8%) after identification.

**TABLE 5:** Characteristics of foodborne illness outbreak investigations — National Environmental Assessment Reporting System, 16 state and local health departments, 2014–2016

Investigation characteristic	No. (%)*
**No. of visits needed to complete the environmental assessment**	
1	202 (49.9)
2	104 (25.1)
3	51 (12.3)
4	26 (6.3)
≥5 (up to 30 visits)	27 (6.5)
**Total**	**410 (100.0)**
**Time interval between establishment identification for an environmental assessment and first contact with the establishment**	
Same day^†^	285 (68.7)
1–2 days	97 (23.4)
3–7 days	24 (5.8)
8–14 days	6 (1.4)
>14 days (up to 36 days)	3 (0.7)
**Total**	**415 (100.0)**
**Time interval between establishment identification for an environmental assessment and establishment observation**	
Same day	175 (49.6)
1–2 days	99 (28.0)
3–7 days	43 (12.2)
8–14 days	18 (5.1)
>14 days (up to 103 days)	18 (5.1)
**Total**	**353 (100.0)**
**Time interval between establishment identification for an environmental assessment and establishment manager interview**	
Same day^†^	82 (25.8)
1–2 days	62 (19.5)
3–7 days	41 (12.9)
8–14 days	27 (8.5)
15–21 days	23 (7.2)
22–28 days	18 (5.7)
29–35 days	18 (5.7)
>35 days (up to 389 days)	47 (14.8)
**Total**	**318 (100.0)**

* Denominators vary because of missing data. Because of rounding, some percentages might not total 100%.

^†^ Includes one situation in which preliminary information led investigators to contact the establishment or conduct a manager interview before the establishment officially was identified for an environmental assessment.

## Discussion

Approximately 60% of foodborne illness outbreaks in retail food establishments reported to NEARS were caused by norovirus, and contamination of food by workers who were ill or infectious contributed to more than half of outbreaks with contributing factors. These findings are similar to national outbreak data reported to FDOSS; a recent analysis found that approximately half of restaurant-associated foodborne illness outbreaks were caused by norovirus, and that workers who were ill or infectious contributed to about half of restaurant-associated outbreaks (*16*). NEARS and FDOSS data both highlight the role of workers in norovirus outbreaks (*2*,*15*,*16*). The data also indicate the need for continued focus on reducing viral foodborne illness outbreaks by protecting food from worker contamination through proper hand hygiene, including glove use, and preventing workers who are ill or infectious from working (*16*,*17*).

NEARS is the only available data source that includes characteristics of retail establishments with foodborne illness outbreaks. Because ill workers are a frequent contributor to outbreaks (*1*), of particular interest are NEARS data on establishment characteristics that might be related to ill worker behavior, such as requiring gloves and excluding workers who are ill from work. Most establishments with outbreaks had policies requiring food workers to wear gloves when handling ready-to-eat foods and preventing those who are ill from working. The FDA Food Code recommends these policies to protect against outbreaks (*2*), yet establishments with these policies still had outbreaks. One reason might be that existing policies are not enforced.

This report assessed whether the establishments had these policies but did not assess whether the policies were regulatory requirements in the areas where the establishments were located. If policies are not regulatory requirements, regulatory officials do not assess them in their inspections and establishments do not receive violations for a lack of policies. Lack of regulation might affect policy effectiveness.

Finally, the mode of the policy might have a role in effectiveness; research suggests that written policies are more effective than unwritten ones (*11*). Written food safety policies might indicate prioritization of food safety or institutionalized policies and practices. Approximately half, or fewer, of the establishments with outbreaks had these policies in writing.

Paid sick leave also might be relevant to outbreaks caused by ill workers; a study found an association between supportive paid sick leave regulations and decreased foodborne illness rates (*18*). Workers have reported that lack of paid sick leave factors into their decision to work while ill (*19*). The relative lack of sick leave for workers suggests this might be a risk factor for foodborne illness outbreaks.

Most outbreaks reported to NEARS occurred in establishments that engaged in complex food preparation processes, served American-style food, were independently owned, and received critical violations on their last inspection. These data can contribute to generating hypotheses about the context in which outbreaks occur. For example, the proportion of establishments with outbreaks engaging in complex food preparation processes (87%) is high compared with the proportion of establishments without outbreaks engaging in these processes (approximately 50%) found in other studies (EHS-Net restaurant cooling practices study, unpublished data, CDC, 2009) (*20*). This difference suggests that outbreaks might occur more often in establishments where complex food preparation occurs. On the other hand, comparisons of establishment ownership indicate that the proportion of independently owned establishments in the NEARS outbreak data set is similar to the proportion of independently owned restaurants nationwide (73% versus 66%) (*21*), which suggests that independent and chain restaurants might experience outbreaks with similar frequency. Although research comparing establishments with and without outbreaks is necessary to confirm these hypotheses, preliminary comparisons indicate the potential value of NEARS data to facilitate development and testing of hypotheses about the characteristics of outbreaks associated with retail food establishments.

NEARS also provides new data that might identify strengths and weaknesses of investigation practices. For example, for most outbreaks the investigators initiated an environmental assessment within a day of learning about the outbreak, which is a positive indicator because experts recommend initiating environmental assessments as quickly as possible (*15*). Research also indicates that timely and comprehensive environmental assessments are associated with identifying factors contributing to outbreaks, which is an important goal of outbreak investigations (*14*). On the other hand, for certain outbreaks, investigators took considerably longer (from 8 days to >14 days) to initiate contact, suggesting a need for improvement in timeliness of environmental assessments. CDC provides free, interactive training on outbreak environmental assessments, a first step for health departments seeking to improve investigation practices (*22*). The CDC-funded Integrated Food Safety Centers of Excellence also provide free resources for food safety professionals (*23*).

## Limitations

The findings in this report are subject to at least four limitations. First, the findings are determined from data reported by a limited number of state and local health departments. Although these health departments represent geographically diverse areas, the foodborne illness outbreaks reported to NEARS are not representative of all U.S. outbreaks. Second, not all outbreaks are identified, reported, or investigated; therefore, the extent to which the outbreaks reported to NEARS represent all outbreaks that occurred in the reporting areas is unknown. Third, outbreak investigation procedures and practices vary across state and local health departments, possibly resulting in systematic differences in data collection. Finally, the manager interview data might be subject to social desirability bias, in which respondents overreport socially desirable conditions, such as the existence of food safety policies in their establishments.

## Future Directions

Most (83.7%) foodborne illness outbreaks reported to NEARS also were reported to FDOSS. Therefore, the data from the two systems can be matched by outbreak to create a comprehensive outbreak data set with epidemiologic, laboratory, and environmental health data. Subsequent analyses of matched data can help guide and develop outbreak prevention efforts. For example, analysis of the relation between establishment policies (environmental health data) and outbreak size (epidemiologic data) can help identify effective policies. Future analyses also might focus on differences between outbreaks that are reported to both NEARS and FDOSS and outbreaks that only are reported to FDOSS. NEARS data also allow comparisons of establishments that have had bacterial outbreaks with those that have had viral outbreaks, which can identify characteristics and policies that might contribute to the likelihood of specific types of outbreaks.

## Conclusion

NEARS provides unique data on establishments that have had foodborne illness outbreaks. These data increase knowledge about the environmental context of outbreaks and contribute to generating hypotheses about their causes and prevention. Use of NEARS data to compare characteristics of establishments with and without outbreaks, examine relations between establishments and epidemiologic characteristics, and compare bacterial and viral outbreaks will contribute to understanding the role of these factors in outbreaks. CDC is developing these analyses, and the information gained from them can help public health authorities develop data-based, effective approaches to prevention of foodborne illness outbreaks. Because NEARS data identify gaps in food safety policies and practices and types of establishments vulnerable to outbreaks, the data also can help target training and interventions for state and local food safety programs and the retail food establishment industry. (For example, the data suggest that outbreaks occur more often in establishments using complex food preparation.) Finally, NEARS data can identify gaps in environmental health investigation practices, such as delayed environmental assessments. Identifying these gaps can help investigators target their improvement efforts, which might include increasing communication among environmental health, epidemiologic, and laboratory programs, as well as implementing policies and training to support environmental assessments (*24*).
